# Micropeptides Encoded in Transcripts Previously Identified as Long Noncoding RNAs: A New Chapter in Transcriptomics and Proteomics

**DOI:** 10.3389/fgene.2018.00144

**Published:** 2018-04-25

**Authors:** Fouzia Yeasmin, Tetsushi Yada, Nobuyoshi Akimitsu

**Affiliations:** ^1^Isotope Science Centre, The University of Tokyo, Tokyo, Japan; ^2^Department of Bioscience and Bioinformatics, Kyushu Institute of Technology, Fukuoka, Japan

**Keywords:** lncRNAs, TUFs, sORFs, micropeptides, translation

## Abstract

Integrative analysis using omics-based technologies results in the identification of a large number of putative short open reading frames (sORFs) with protein-coding capacity within transcripts previously identified as long noncoding RNAs (lncRNAs) or transcripts of unknown function (TUFs). sORFs were previously overlooked because of their diminutive size and the difficulty of identification by bioinformatics analyses. There is now growing evidence of the existence of potentially functional micropeptides produced from sORFs within cells of diverse species. Recent characterization of a few of these revealed their significant divergent roles in many fundamental biological processes, where some also show important relationships with pathogenesis. Recent works therefore provide new insights for exploring the wealth of information that may lie within sORF-encoded short proteins. Here, we summarize the current progress and view of micropeptides encoded in sORFs of protein-coding genes.

## Introduction

Identification of a large number of RNA transcripts by genome-wide analysis suggests a complex network of transcripts that includes tens of thousands of long noncoding RNAs (lncRNAs) and transcripts of unknown function (TUFs) (Carninci et al., 2005; Willingham et al., 2006; Birney et al., 2007; Kapranov et al., 2007). Recent studies have suggested that lncRNAs and TUFs in the human genome represent the greatest source for short open reading frames (sORFs), which were previously overlooked because of their small size and the lack of evidence for “codingness” (Frith et al., 2006; Cohen, 2014; Pauli et al., 2015). As a result, sORFs embedded in lncRNAs and TUFs have not been adequately studied.

sORF-encoded micropeptides first attracted the attention of a group of scientists during their study of lncRNA (Rohrig et al., 2002). From that point, many studies have been carried out to identify potential sORF candidates, and whether there are any more of them that can encode functional micropeptides. Recent advancements in bioinformatics, proteomics and transcriptomics have revealed that traditional computational algorithms used in searches for many potent ORFs may have included oversights as many studies have now identified hundreds of non-annotated sORFs that have coding potential for micropeptides (Ingolia et al., 2011; Slavoff et al., 2013; Bazzini et al., 2014) from yeast (Smith et al., 2014) to plants (Hanada et al., 2013; Lauressergues et al., 2015) and humans (Ingolia et al., 2014; Ma et al., 2014). sORF-encoded proteins have emerged as a new, functional class because of their role in many biological activities (Crappé et al., 2014). The diverse biological functions of this new group of short proteins have attracted the attention of the scientific community and increased interest in studying them in more detail (Saghatelian and Couso, 2015; Makarewich and Olson, 2017).

Here, we give a brief overview of the various approaches recently used to identify sORF- encoded micropeptides and their biological function. Based on the results of previous studies, we also try to identify the potential ideas and strategies that can be implemented to characterize other micropeptides' functionalities. Finally, we review the diverse biological function of micropeptides that have been found up until recently, from plants to animals. These suggesting that many biologically significant micropeptides may be concealed in the hidden world of proteomes.

## More developed techniques identify more potent sORF-encoded micropeptides

Traditional computational prediction of protein-coding ORFs relies on a number of stringent criteria to remove meaningless ORFs, such as size cutoff of 300 nucleotides, AUG start codon usage, and sequence conservation (Gish and States, 1993; Kochetov, 2005), rendering them inappropriate for sORF detection. Hunting for these tiny treasures has therefore posed a great challenge.

However, with the advancement of technology, the challenge has begun to be addressed effectively. Both computational and experimental approaches have made it easier to explore the complexity of the small proteome. Several approaches have been taken to systematically annotate sORFs with coding potential. Along with other conventional strategies, such as cross-species comparison, examination of codon content and coding features used to identify ORFs, various metrics and methods have been developed and are playing prominent roles in identifying putative sORFs (Table 1).

**Table 1:** Computational and experimental approaches to protein-coding sORFs.

**Metrics and methods to identify sORF (including both computational and experimental)**		**Description**	**References**
Computing-based method	sORFfinder, HAItORF, uPEPperoni	Web based tools to locate sORF having coding potential	Hanada et al., 2010; Vanderperre et al., 2012; Skarshewski et al., 2014
	PhyloCSF	A computational method examines evolutionary conservation of a sORF across species	Lin et al., 2011
Transcriptomic-based method	Ribosome profiling	A deep sequencing- based tool of ribosome protected mRNA fragments to obtain a global snapshot of translation	Ingolia et al., 2011
	Poly-ribo seq	A combination of ribosome profiling and polysome to enrich more potent protein coding ORFs	Aspden et al., 2014
	Ribosome releasing scores (RRS)	These three metrics are developed and combined with ribosome profiling to assist in identification of true protein coding ORFs	Guttman et al., 2013
	Fragment length organization similarity score (FLOSS)		Ingolia et al., 2014
	ORF regression algorithm for translation evaluation RPFS (ribosome-protected mRNA fragments) (ORF-RATER)		Fields et al., 2015
Proteomics-based	Proteo genomics	A combined approach of proteomics and genomics	Slavoff et al., 2013

Ribosome profiling has emerged as a technique for comprehensively and quantitatively measuring translation (Ingolia et al., 2014; Smith et al., 2014). Based on modification of ribosome foot printing, it is mainly premised on deep sequencing of ribosome-protected mRNA fragments to obtain a global snapshot of translation. Application of ribosome profiling has provided several key findings, including prodigious use of non-ATG initiation codons, as well as identification of polycistronic genes, upstream ORFs and overlapping ORFs. Hundreds of putative non-annotated protein-coding sORFs have recently been identified in eukaryotic genomes by using this technique (Ingolia et al., 2011; Bazzini et al., 2014).

However, ribosome occupancy does not always mean true translation, as indicated by the identification of many well-characterized nuclear lncRNAs in a ribosome profiling assay (Brannan et al., 1990; Guttman et al., 2013). Many ORFs are associated with ribosomes to regulate the translation of downstream ORFs. This suggests ribosome profiling is not sufficient evidence of protein synthesis. To differentiate more effective protein-coding transcripts from noncoding RNAs, several algorithms and metrics have been developed based on their ribosome-profiling characteristics, including RRS (Guttman et al., 2013), FLOSS (Ingolia et al., 2014), ORF-RATER (Fields et al., 2015), and Ribo taper (Calviello et al., 2016).

Poly-Ribo-Seq, a modification of a ribosome-profiling method, enriches polysomes that are more likely to be actively translating mRNA into proteins. Poly-Ribo-Seq was successfully used to identify several sORFs in the Drosophila genome (Galindo et al., 2007; Aspden et al., 2014).

Mass spectrometry (MS) peptidomics and proteomics experiments have recently been applied to identify sORF-encoded micropeptides. MS is advantageous compared with ribosome profiling, as it directly detects the peptide generated from ORFs and therefore validates the production of peptides. However, the bias of MS toward more abundant proteins means it only detects the peptides abundant in cells. Analysis of tandem mass spectrometry (MS/MS) data that mapped expressed peptides to their encoding genomic loci and transcriptome data generated by ENCODE has identified 85 unique peptides that match with 69 lncRNAs (Bánfai, 2012). Slavoff et al. developed a modified proteomic strategy, known as proteogenomics to identify and validate more potent sORFs, wherein they compiled a custom mRNA-seq derived polypeptide database to identify MS fragmentation spectra. In this approach, the proteome is enriched to isolate small polypeptides before proteomic analysis. Through this strategy, 86 uncharacterized SEPs (sORF-encoded polypeptides) of 90 were identified in K562 cells (Slavoff et al., 2013). There are also still some difficulties to consider. The average tissue content of micropeptides is very low, and they are often subjected to degradation or loss during sample preparation, which further impedes their identification. As a result, many micropeptides produced in cells may be absent in MS analysis. New and alternative extraction methods may prove more effective in extracting and identifying micropeptides. For example, Schwaid et al. described an affinity-based approach that could enrich and identify cysteine-containing human sORF-encoded polypeptides (ccSEPs) in cells. They were able to identify 16 novel sSEPs from previously uncharacterized sORFs (Schwaid et al., 2013). MS-based methods have thus, to date, identified a limited number of micro-proteins.

## sORF-encoded micropeptides: insights into their function

Small peptides have high recognition because of their important roles in diverse biological processes (Fricker, 2005; Boonen et al., 2009; Cabrera-Quio et al., 2016). The largest and most extensively studied class of small peptides are classical bioactive peptides, which are derived from larger precursor proteins and contain N-terminal signal sequences. Hormones and neuropeptides are considered the best examples of bioactive molecules (Hashimoto et al., 2001; Cunha et al., 2008). Most of these peptides act as ligands of membrane receptors (Boonen et al., 2009). Micropeptides differ from these bioactive small peptides in that they are not processed from large peptides but rather are translated from sORFs previously identified as lncRNAs and TUFs. Four initial studies (Rohrig et al., 2002; Savard et al., 2006; Galindo et al., 2007; Kondo et al., 2007) were pioneering in opening up new avenues for sORF research. Their studies showed how a sORF can be involved in different developmental contexts with apparently different biological roles during morphogenesis.

**Figure 1:**
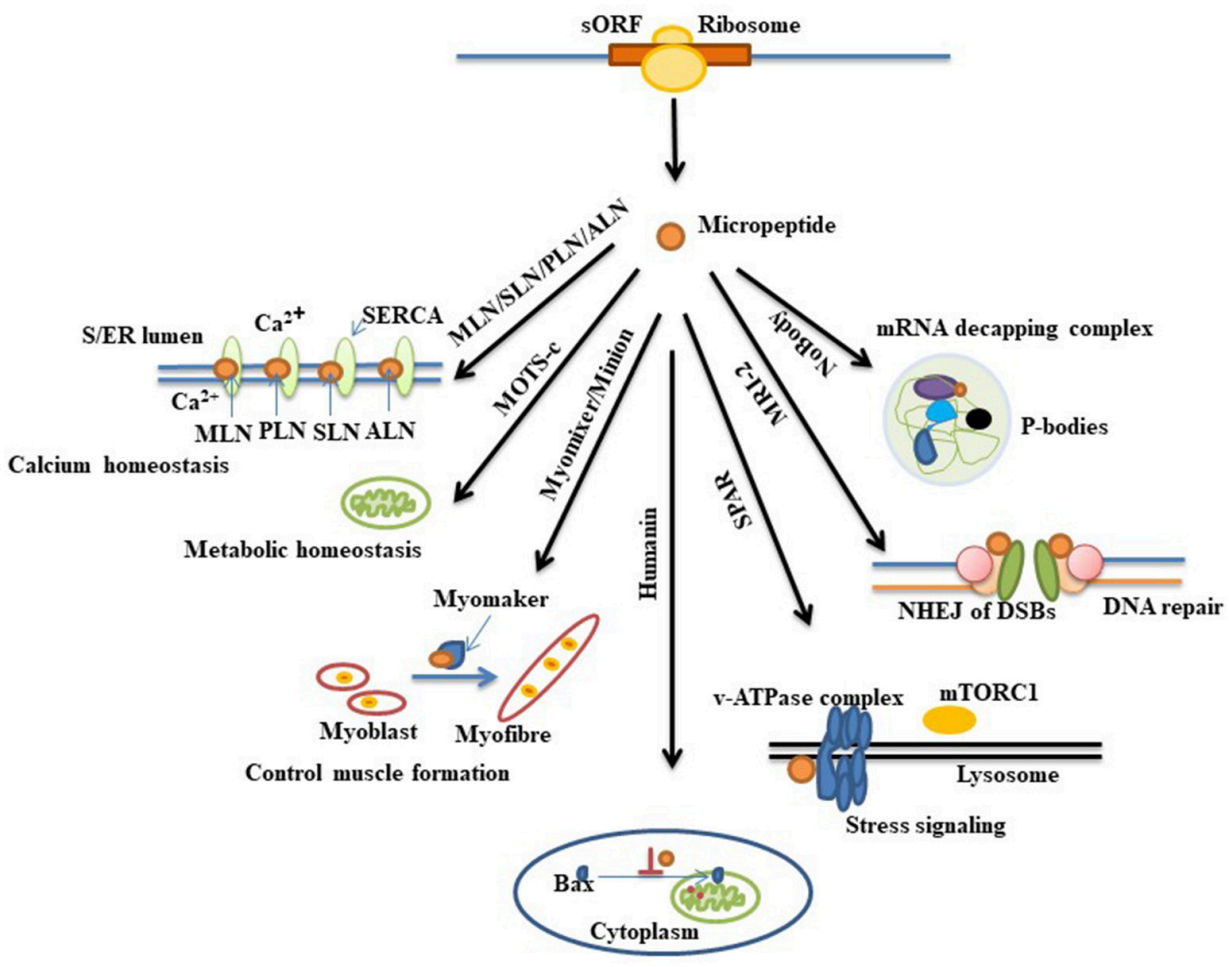
Diverse biological function of recently annotated micropeptides. Micropeptides are found to be involved in many biological processes. Myoregulin (MLN), phospholamban (PLN), sarcolipin (SLN), and another regulin (ALN) are a group of peptides that interact with the protein SERCA (a Ca^2+^ Pump) in sarcoplasmic and endoplasmic reticulum (S/ER) and maintain Ca^2+^ homeostasis in the cell. MOTS-c and humanin are mitochondrial sORF-encoded micropeptides that display important roles in metabolic homeostasis and apoptosis, respectively. Humanin suppresses apoptosis by preventing the translocation of an apoptosis inducing protein, Bax (Bcl2-associated X protein), from cytoplasm to mitochondria. Another micropeptide named MRI-2 is found to enhance non-homologous end joining (NHEJ) of double-strand DNA breaks (DSBs) by associating with other DNA end-binding proteins (Ku proteins). Myomixer, minion, SPAR, and NoBody, four other micropeptides that have been recently discovered, have distinct biological roles wherein myomixer and minion stimulate the fusion of myoblast to form myofiber during muscle formation by participating with another protein, myomaker. The micropeptide SPAR is localized into lysome where it interacts with the lysosomal v-ATPase complex and regulates mTORC1 protein activation during stress signaling. NoBody, a p-body (processing-body, which is involved in mRNA turnover) dissociating micropeptide, shows its function by interacting with the mRNA decapping complex.

**Figure 2:**
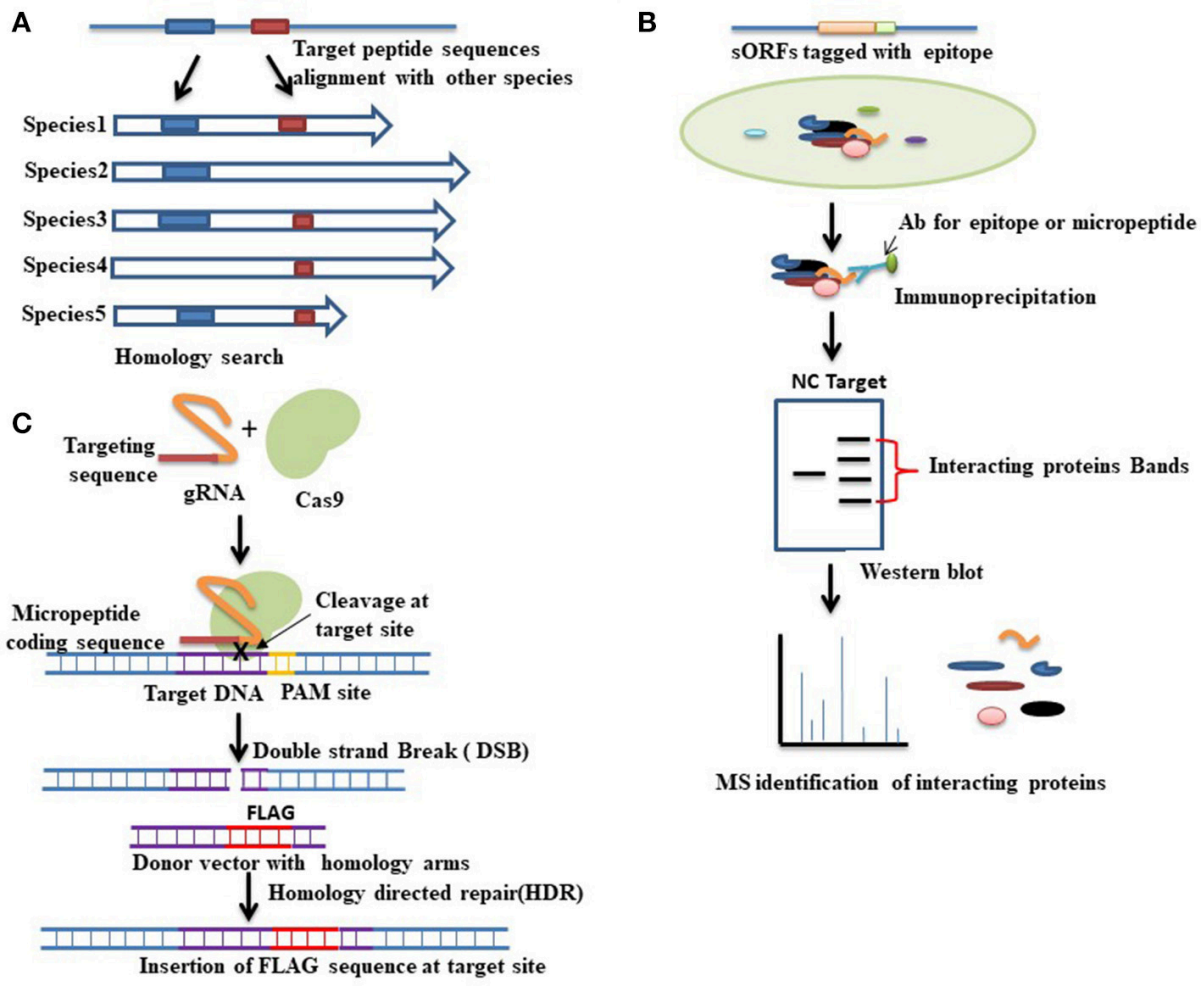
Various approaches for functional characterization of micropeptides. **(A)** Evolutionary conservation of a peptide sequence is suggestive of functionality. Homology- based searching among species thus can be performed to identify whether the target peptide sequence shares any functional similarity with other proteins. Here the blue and red boxes indicate the conserved sequences among species. **(B)** Functional proteomics is a commonly used approach for identifying the interacting proteins of a target protein. In this method, first, immunoprecipitation is conducted by using an antibody (Ab) that is designed either against the epitope tagged with a target micropeptide or directly against the micropeptide. Western blot is then performed followed by mass spectrometry analysis to separate and identify the interacting proteins. Red brackets indicate the bands of interacting proteins that are separated by western blot analysis. A negative control (NC) denotes an empty vector that also runs for comparison. The nature of the interacting protein will thus provide clues about the function of the target micropeptide. **(C)** CRISPR-cas9 mediated gene editing approaches can also be used to check the coding potential of sORFs. To verify the coding potential, an epitope tag (FLAG) can be inserted at the downstream of the sORF into the endogenous locus. CRISPR-cas9 mediated gene editing is started by the recognition of the target site, which is mediated by a guide RNA (gRNA). Guide RNA guides the cas9 endonuclease to a specific location in the genome sequence, which is immediately adjacent to a protospacer adjacent motif (PAM). Upon recognition, the cas9 creates a double strand break (DSB) at the target site. This DSB can then be repaired either by non-homologous end joining (NHEJ) or by homology directed repair (HDR). HDR is used to insert an epitope tag at the target site where a donor vector with homology to the targeted locus must be provided. The donor vector must contain the epitope tag that has to be knocked-in at the target site. Expression of the engineered fusion protein can then be verified by western blot analysis.

As described above, advancements in technologies over the past few years have led to the discovery of several hundred of putative coding sORFs in various species. However, it is still unknown how many of these newly discovered sORF-encoded peptides are functional. Existence of a peptide does not always imply it has a function. Experimental demonstration is important in revealing their biological effects. Several approaches can be used to validate candidate-translated sORFs (Housman and Ulitsky, 2016). Recently some micropeptides have been characterized and found to play important roles in fundamental biological processes such as RNA decapping (D'Lima et al., 2017), DNA repair (Slavoff et al., 2014), stress signaling (Matsumoto et al., 2017), apoptosis (Guo et al., 2003), muscle formation (Bi et al., 2017), metabolic homeostasis (Lee et al., 2015), and calcium homeostasis (Magny et al., 2013; Anderson et al., 2015, 2016; Nelson et al., 2016; Figure 1).The following section briefly explains commonly used strategies for deciphering the functions of short proteins that are necessary for their characterization (Figure 2).

## *in silico* (or computational) characterization

Evolutionary conservation is an important sign that a gene is functional. One hallmark of the sORFs studied thus far is evolutional conservation of micropeptides. An evolutionary conserved micropeptide called *polished rice* (*pri*) or *tarsal-less* (tal) was identified in *Drosophila*, while the *Tribolium* orthologue is known as *mille-pattes (mlpt)* (Savard et al., 2006; Galindo et al., 2007; Kondo et al., 2007). These micropeptides were characterized based on their conservation. Homology-based searching among species for unannotated micropeptides may be performed to predict any conserved biological function (Figure 2). The best example of homology-based characterization is the identification of a group of micropeptides, namely, myoregulin (MLN), phospholamban (PLN), and sarcolipin (SLN). They share conserved peptide sequences from flies to vertebrates involved in Ca^2+^ homeostasis through inhibiting SERCA activity (Magny et al., 2013) in muscle. There is a sequence and structural similarity among these peptides. Later, another two micropeptides, endoregulin (ELN), and another-regulin (ALN), were also characterized based on their shared amino acids, and found to show similar functions to MLN/PLN/SLN, but in nonmuscle cell types (Anderson et al., 2016).

Thus, identification and characterization based on sequence features is a reasonable approach for deciphering the biological function of new unannotated micropeptides. Computational predictions of functional sORFs use several key features to identify potential sORFs. Canonical protein-coding ORFs show striking sequence features as measured by the ratio of Ka and Ks (Ka/ Ks < 1, the ratio of synonymous versus nonsynonymous codon substitution), suggesting that canonical protein coding genes are under selective pressure during evolution. Compared with canonical protein coding genes, it is difficult to score statistically significant values for very short sequences because the number of possible changes is low (Ladoukakis et al., 2011). Mackowiak and his group brought a new computational approach to identify conserved sORFs using comparative genomics (Mackowiak et al., 2015). Three qualitative features of coding sequence conservation specific to known micropeptides and canonical proteins were analyzed in their study. The first is the conservation of amino acid sequences by phylogenetic codon substitution frequencies (PhyloCSF). Second is the conservation of the reading frame, which is the conservation of in-frame start and stop codons in related species. The third is a drop in nucleotide sequence conservation around the start and stop codons using PhastCons (Siepel et al., 2005). The combination of these three features has identified about 2,000 sORFs in five systems: human, mouse, zebrafish, fruit fly, and the nematode Caenorhabditis elegans. Translation and protein expression of some of these predicted sORFs have also been confirmed by experimental evidence.

Although functional characterization of sORFs based on sequence conservation is useful, it is not applicable for all. Some non-conserved sORFs may evolve as newly coding ORFs that can also be present and be involved with regulatory functions.

## Functional proteomics

Although some sORFs are found to be highly conserved across species, most show relatively low sequence conservation compared with known protein-coding genes (Carvunis et al., 2012; Slavoff et al., 2013). Therefore, although homology-based functional characterization is reasonable, as mentioned above, it has difficulty finding species-specific functional peptides. Several of the micropeptides characterized thus far exert their functions by interacting with other proteins. Several studies have applied functional proteomics successfully to identify the interacting partners. For example, Matsumoto and colleagues employed functional proteomics to study a LINC00961-encoded short protein. This micropeptide interacts with the lysosomal v-ATPase complex to regulate mTORC1 (a rapamycin protein complex) activation (Figure 1) and muscle regeneration. This interaction with the v-ATPase complex and regulation of mTORC1 is specific to the amino acid response. It is therefore known as a small regulatory polypeptide of the amino acid response, or SPAR (Matsumoto et al., 2017).

By employing functional proteomics, another group also characterized and identified the biological significance of another unreported micropeptide, named NoBody (D'Lima et al., 2017). By performing immunoprecipitation and MS analysis, the researchers found NoBody to be a component of the mRNA decapping protein complex that cross-links to EDC4 (enhancer of mRNA decapping 4). The mRNA decapping complex removes the 5′ cap from mRNAs to promote 5′-3′ decay. Molecular components of this pathway localize to p-bodies. Manipulation of NoBody expression is anticorelated with the P-body number. NoBody regulates the P-body number in cells by interacting with decapping proteins. This micropeptide is therefore called the non-annotated P-body dissociating polypeptide (NoBody).

However, traditional immunoprecipitation methods very often result in the enrichment of many nonspecific interactions of micropeptides. For example, functional proteomics analysis of a micropeptide named modulator of retroviral infection (MRI) has revealed that it is associated with ku70 and ku80, two essential proteins that are involved in the nonhomologous end joining DNA repairing mechanism (Slavoff et al., 2014). Association of MRI with ku70/ku80 suggests that it is involved in the cellular DNA repairing mechanism. Although the immunoprecipitation of MRI also enriched for heat shock protein 70 family members protein, imaging studies ruled out cytosolic heat shock proteins as bona fide interactors that might be formed after the cells are lysed during the immunoprecipitation (Slavoff et al., 2014; Grundy et al., 2016). Such a problem thus demands a better approach for identifying micropeptide associated proteins and protein complexes. Recently Chu and colleagues applied an in-situ proximity tagging method to elucidate microprotein-protein interactions (MPIs) for an uncharacterized microprotein called c11orf98 (Chu et al., 2017). This method relies on an engineered ascorbate peroxidase (APEX) (Rhee et al., 2013). When APEX fusion protein is expressed in the cells and treated with hydrogen peroxide (H_2_O_2_) in the presence of biotin-phenol, the proteins proximal to the APEX fusion protein are labeled with biotin. The proteins, that are biotinylated, can then be enriched and analyzed by MS. Thus, the analysis of biotinylated proteins provides valuable information about the protein environment of fusion protein. Since the interactions take place in the context of a living cell, the enrichment of nonspecific interactors is reduced. By applying this approach, it was revealed that c11orf98 interacts with nucleolar proteins nucleoplasm and nucleolin (Chu et al., 2017), which suggests that the application of APEX tagging is useful to characterize uncharacterized micropeptides.

These studies suggest that functional proteomics may be implemented to understand the function and biological nature of an unannotated short protein through identifying direct binding partners or components (Figure 2).

## Gene editing approaches

Recently developed Clustered regularly interspaced short palindromic repeats (CRISPR)-associated protein (cas9) mediated gene editing technology has become a powerful approach among scientists to study a gene's function. CRISPR-cas9 mediated gene editing strategies can also be used for identifying and verifying coding potential of sORF encoded peptides. An epitope tag can be knocked-in into the endogenous locus of a micropeptide in-frame with the predicted sORF to produce a fusion protein using CRISPR/cas9-mediated homologous recombination (Figure 2). Detection of the engineered fusion protein by western blot analysis provides the evidence that the mRNA is translated into a stable peptide. This powerful knock-in technique also simplifies many downstream applications that are important for functional characterizing of a gene. For example, immunoprecipitation to identify binding partners of the target proteins. Immunocytochemistry can also be performed in epitope-tagged samples to check the subcellular localization of the fusion protein, which may provide important information about its involvement in biological processes. Recently some research groups have implemented this new technology to verify sORF-encoded peptides (Galindo et al., 2007; Slavoff et al., 2014; Anderson et al., 2015). By using CRISPR-cas9 homologous recombination, an epitope tag was inserted at the downstream of the sORF to confirm whether the sORF containing gene was actively transcribed from its native chromosomal context and translated into a stable peptide. Identification and validation of some sORF-encoded peptides by CRISPR-cas9 mediated gene editing technologies thus indicate the possible successful application of them in identifying and verifying other sORF-encoded peptides.

## Diverse biological functions of micropeptides

### In plants

The first eukaryotic micropeptide was identified in plants by a group of researchers studying legumes. A gene called early nodulin 40 (Enod40), previously annotated as lncRNA, was found to encode two short peptides of 12 and 24 amino acids (AAs) in plants, where they interact with a sucrose-synthesizing enzyme during root nodule organogenesis (Rohrig et al., 2002). Since the discovery of the first micropeptide in plants, others have also been functionally characterized. The 36 AAs peptide, which is encoded by the POLARIS (PLS) gene in *Arabidopsis*, has been shown to affect root growth and leaf vascular patterning (Casson et al., 2002; Chilley et al., 2006). Another two micropeptides, 76 AAs Brick1 (Brk) and 53 AAs ROTUNDIFOLIA (ROT4), were also found to be involved with leaf morphogenesis. In maize, the recessive mutation of Brk1 results in several morphological defects of leaf epithelia (Frank and Smith, 2002). However, ROT4 regulates polar cell proliferation in lateral organs and leaf morphogenesis in *Arabidopsis* (Narita et al., 2004). In *Arabidopsis*, two other best-characterized micropeptides were reported: a 51 AAs ROT18/DLV1 and a 25 AAs kiss of death (KOD), which are involved in plant organogenesis (Wen et al., 2004; Valdivia et al., 2012; Guo et al., 2015) and programmed cell death regulation (Blanvillain et al., 2011), respectively. Recently two newer micropeptides have also been identified in maize, Zm401p10 and Zm908p11 with 89 and 97 AAs, respectively, which are involved in pollen development (Ma et al., 2008; Wang et al., 2009; Dong et al., 2013). Characterizations of these micropeptides indicate their functional diversity ranging from plant development to growth, nodulation, organogenesis, pollen development, and cell death.

### In animals

The first identification of micropeptides in animals came from the study of lncRNAs in Drosophila. The sORFs of the long noncoding RNA, namely, polished rice or tarsal-less (tal), encode four micropeptides from 11 to 32 AAs are required during the embryonic development of flies (Galindo et al., 2007; Kondo et al., 2007, 2010). By triggering proteasome-mediated protein processing, the *pri* micropeptide converts a transcription factor, shavenbaby (Svb), from a repressor into an activator (Zanet et al., 2015). Since then, a handful of micropeptides have been functionally characterized (Table 2). To identify the characterizing signal molecules from the nonannotated translated sORFs, the Pauli group identified a micropeptide, Toddler, which acts as a motogen, a signal that promotes cell migration. Toddler activates G-protein-coupled APJ (apelin) signaling for this function (Pauli et al., 2014). AGD3, previously classified as a TUF, encodes a small protein of 63 AAs and has been found to show involvement in human stem cell differentiation (Kikuchi et al., 2009). Recently a group of micropeptides was found to show a prominent role in calcium homeostasis, both in skeletal and nonskeletal muscle cells, through the binding and inhibiting of a well-known Ca^2+^ ATP- ase pump, SERCA, thereby influencing regular muscle contraction (Magny et al., 2013; Anderson et al., 2015). Nelson et al. described the opposite activity of another lncRNA-derived micropeptide in mammalian muscle, called DWORF (dwarf open reading frame). This micropeptide enhances SERCA activity by displacing those inhibitory proteins and boosts muscle performance. DWORF is abundantly expressed in the mouse heart, and is suppressed in ischemic human heart tissue, suggesting a possible link with heart failure (Nelson et al., 2016). Myomixer, a micropeptide of 84 AAs also has a function in the muscle but is unlike DWORF or other micropeptides in this group. Myomixer plays a role in controlling muscle formation by associating with a fusogenic membrane protein, myomaker, and favors formation of multinucleated myofibers in mice (Bi et al., 2017). Recently, another peptide known as minion (microprotein inducer of fusion), which is specific for skeletal muscle, has been identified. Functional characterization of this microprotein revealed that like myomixer, minion also controls cell fusion, and muscle formation by associating with myomaker (Zhang et al., 2017). The functionality of micropeptides has also been found in the DNA repairing process. For example, a 69 AAs small peptide, MRI-2, has been identified as a novel factor of the non-homologous end join factor (NHEJ). MRI-2 stimulates NHEJ by interacting with Ku protein, a DNA end-binding protein (Slavoff et al., 2014). As more micropeptides are characterized, more hidden functions are unfolded, as exemplified by another micropeptide that is encoded by a putative lncRNA HOXB-AS3. This conserved 53 AAs peptide, HOX-AS3, inhibits tumorigenesis by the regulation of PKM alternative splicing and metabolic reprogramming of colon cancer cells (Huang et al., 2017). NoBody and SPAR are two additional examples of functional micropeptides, which as we described above, have been characterized recently by their distinct biological significance.

**Table 2:** Micropeptides and their diverse biological functions.

**Origin**	**Micro-peptides**	**Conservation**	**Method of identification/characterization**	**Function**	**Size (AAs)**	**References**
Plant	Early nodulin 40 (Enod 40)	Plants	*In vitro* translation	Nodule organogenesis	12.24	Rohrig et al., 2002
	POLARIS (PLS)		Gene expression analysis by promoter trapping; Mutation analysis	Leaf morphogenesis	36	Casson et al., 2002; Chilley et al., 2006
	Brick1 (Brk)	Plants and animals	Mutation analysis	Leaf morphogenesis	76	Frank and Smith, 2002
	ROTUNDIFOLIA (ROT4)	Plants	Screening of a mutant in *Arabidopsis thaliana*	Leaf morphogenesis	53	Narita et al., 2004
	ROT18/DLV1	Plants	Gain of function screening of genes responsible for fruit growth and development in Arabidopsis	Plant organogenesis	51	Wen et al., 2004; Guo et al., 2015
	Kiss of death (KOD)		Gene expression analysis by promoter trapping	Programmed cell death regulation	25	Blanvillain et al., 2011
	Zm401p10, Zm908p11	Poaceae	Bioinformatics analysis	Pollen development	89.97	Ma et al., 2008; Wang et al., 2009; Dong et al., 2013
Animal	Polished rice (Pri)	Insects	Mutation analysis	Fly embryogenesis	11–32	Galindo et al., 2007; Kondo et al., 2007, 2010
	Toddler	Vertebrates	Ribo-seq-based search for novel signaling peptides	Promotes cell migration	58	Pauli et al., 2014
	AGD3	Mammals	Sequencing analysis.	Involve in stem cell differentiation	63	Kikuchi et al., 2009
	Myoregulin (MLN)	Mammals	Bioinformatics approaches; Homology-based characterization	Calcium homeostasis	46	Magny et al., 2013; Anderson et al., 2015
	DWORF	Lamprey	PhyloCSF search; Gain and loss of function	Enhance muscle performance	34	Nelson et al., 2016
	Myomixer	Vertebrates	CRISPR-cas9 mediated loss of function screening of genes required for myoblast fusion	Functionally involve in controlling muscle performance	84	Bi et al., 2017
	MRI-2	Mammals	HPLC-MS/MS screening combining with RNA seq; characterized by functional proteomics	DNA repairing process	69	Slavoff et al., 2014
	NoBody	Mammals	HPLC-MS/MS screening combining with RNA seq; characterized by functional Proteomics	mRNA recycling	68	D'Lima et al., 2017
	SPAR	Human and mouse	Proteomics strategy	Regulate muscle regeneration	90	Matsumoto et al., 2017
	Humanin	Different species	Functional expression screening	Involve in program cell death	24	Hashimoto et al., 2001; Guo et al., 2003
	MOTS-c	14 species	*In silico* search for potential sORFs in human 12srRNA.	Metabolic Homeostasis	16	Lee et al., 2015
	Minion	Mammalian species	RNA seq analysis of uninjured and regenerating muscle	Muscle formation	84	Zhang et al., 2017
	HOXB-AS3	Primates	Ribosome profiling	Suppresses colon cancer growth	53	Huang et al., 2017

According to Weissman, some micropeptides might also be immunogenic without a clear functional role. For example, micropeptides derived from human-infecting cytomegalovirus (HCMV) lncRNA β2.7, were found to robustly stimulate T cell memory responses only in humans with a history of HCMV infection (Fields et al., 2015). Very recently, another group of scientists identified some micropeptides that exhibited differential regulation upon viral infection (Razooky et al., 2017). These indicate that there may be more sORFs that are involve with certain diseases. Thus, translation of some ORFs that have been previously overlooked may contribute in important ways to cell biology.

Biologically significant micropeptides are not only found to be encoded by nuclear-encoded transcripts. Mitochondrial genomes also contribute in the proteome by producing biologically important micropeptides. Humanin, a signaling peptide encoded by mitochondrial sORFs, is functionally involved with programmed cell death. It inhibits translocation of an apoptosis-inducing protein, Bax (Bcl2-associated x-protein), from cytoplasm to mitochondria, and thereby regulates apoptosis (Guo et al., 2003). Humanin also shows neuroprotective effects and is known as a peptide against neurotoxicity related diseases (Matsuoka et al., 2006). Another micropeptide of 16 AAs was also found to be encoded by mitochondrial 12sRNA, named MOTS-c. MOTS-c shows endocrine-like effects on muscle metabolism, insulin sensitivity and weight regulation (Lee et al., 2015). Identification of the mitochondrial-encoded peptides humanin and MOTS-c suggests the possible existence of more potent sORFs in mitochondria along with their role as regulators of biological processes.

The diverse biological functions of these micropeptides serve as an indication that we are at the very beginning of exploring the mystery of micropeptides.

## Conclusions

Technological advances have uncovered the existence of several hundred putative sORF-encoded micropeptides throughout the genomes. Recent identification and characterization of a small number of sORF-encoded micropeptides and their biological role indicate that there is a hidden world of active peptides waiting to be explored. A great deal of effort is still needed to validate whether each of these peptides is biologically important or if they are just transcriptional/translational noise. Some widely used approaches, such as homology-based functionality search, functional proteomics, gene editing technologies, and massive sequencing-based approach, can be implemented on uncharacterized micropeptides to reveal their biological relevance. Tiny size, low abundance, rapid degradation and loss during sample preparation often make it difficult to work with micropeptides, demanding more sensitive and sophisticated methods. Thus, there are many technical challenges in facilitating the study of micropeptides.

Functional studies of micropeptides in a wide range of species demonstrate that they have important biological functions, including involvement in human pathogenesis. HOXB-AS3, DWORF and humanin are some examples of this group, which show involvement in cancer, heart diseases, and neurotoxicity related diseases, respectively. In addition to these, involvement of a group of newly identified micropeptides against viral infection mediated pathogenesis also suggest that there are more micropeptides that may be involved with certain diseases in humans. These findings indicate that micropeptides may represent new opportunities for drug therapies.

Although some of the micropeptides are functionally characterized, the exact mechanism of their mode of action is unclear. Complete understanding of their action may play an important role in therapeutic purposes, where a drug may be designed by modulating or mimicking their function to regulate any biological pathway they may be involved in.

These recent findings provide new insights into sORF-encoded micropeptides as a new and important class of biological molecules and offer new avenues of research in the proteomics world.

## Author contributions

All authors listed have made a substantial, direct and intellectual contribution to the work, and approved it for publication.

### Conflict of interest statement

The authors declare that the research was conducted in the absence of any commercial or financial relationships that could be construed as a potential conflict of interest.

## References

[B1] Anderson D. M. Anderson K. M. Chang C. L. Makarewich C. A. Nelson B. R. McAnally J. R.. (2015). A micropeptide encoded by a putative long noncoding RNA regulates muscle performance. Cell 160, 595–606. 10.1016/j.cell.2015.01.009 25640239 PMC4356254

[B2] Anderson D. M. Makarewich C. A. Anderson K. M. Shelton J. M. Bezprozvannaya S. Bassel-Duby R.. (2016). Widespread control of calcium signaling by a family of SERCA-inhibiting micropeptides. Sci. Signal. 9:ra119. 10.1126/scisignal.aaj1460 27923914 PMC5696797

[B3] Aspden J. L. Eyre-Walker Y. C. Philips R. J. Amin U. Mumtaz M. A. S. Brocard M.. (2014). Extensive translation of small open reading frames revealed by Poly-Ribo-Seq. Elife 3:e03528. 10.7554/eLife.03528 25144939 PMC4359375

[B4] Bánfai B. Jia H. Khatun J. Wood E. Risk B. Gundling W. E.. (2012). Long noncoding RNAs are rarely translated in two human cell lines. Genome Res. 22, 1646–1657. 10.1101/gr.134767.111 22955977 PMC3431482

[B5] Bazzini A. A. Johnstone T. G. Christiano R. Mackowiak S. D. Obermayer B. Fleming E. S.. (2014). Identification of small ORFs in vertebrates using ribosome footprinting and evolutionary conservation. EMBO J. 33, 981–993. 10.1002/embj.201488411 24705786 PMC4193932

[B6] Bi P. Ramirez-Martinez A. Li H. Cannavino J. McAnally J. R. Shelton J. M.. (2017). Control of muscle formation by the fusogenic micropeptide myomixer. Science 356, 323–327. 10.1126/science.aam9361 28386024 PMC5502127

[B7] Birney E. Stamatoyannopoulos J. A. Dutta A. Guigó R. Gingeras T. R. Margulies E.. (2007). Identification and analysis of functional elements in 1% of the human genome by the ENCODE pilot project. Nature 447, 799–816. 10.1038/nature05874 17571346 PMC2212820

[B8] Blanvillain R. Young B. Cai Y. M. Hecht V. Varoquaux F. Delorme V.. (2011). The Arabidopsis peptide kiss of death is an inducer of programmed cell death. EMBO J. 30, 1173–1183. 10.1038/emboj.2011.14 21326210 PMC3061025

[B9] Boonen K. Creemers J. W. Schoofs L. (2009). Bioactive peptides, networks and systems biology. BioEssays 31, 300–314. 10.1002/bies.200800055 19260025

[B10] Brannan C. I. Dees E. C. Ingram R. S. Tilghman S. M. (1990). The product of the H19 gene may function as an RNA. Mol. Cell. Biol. 10, 28–36. 10.1128/MCB.10.1.28 1688465 PMC360709

[B11] Cabrera-Quio L. E. Herberg S. Pauli A. (2016). Decoding sORF translation - from small proteins to gene regulation. RNA Biol. 13, 1051–1059. 10.1080/15476286.2016.1218589 27653973 PMC5100344

[B12] Calviello L. Mukherjee N. Wyler E. Zauber H. Hirsekorn A. Selbach M.. (2016). Detecting actively translated open reading frames in ribosome profiling data. Nat. Methods 13, 165–170. 10.1038/nmeth.3688 26657557

[B13] Carninci P. Kasukawa T. Katayama S. Gough J. Frith M. C. Maeda N.. (2005). The transcriptional landscape of the mammalian genome. Science 309, 1559–1563. 10.1126/science.1112014 16141072

[B14] Carvunis A. R. Rolland T. Wapinski I. Calderwood M. A. Yildirim M. A. Simonis N.. (2012). Proto-genes and *de novo* gene birth. Nature 487, 370–374. 10.1038/nature11184 22722833 PMC3401362

[B15] Casson S. A. Chilley P. M. Topping J. F. Evans I. M. Souter M. A. Lindsey K. (2002). The *POLARIS* gene of Arabidopsis encodes a predicted peptide required for correct root growth and leaf vascular patterning. Plant Cell 14, 1705–1721. 10.1105/tpc.002618 12172017 PMC151460

[B16] Chilley P. M. Casson S. A. Tarkowski P. Hawkins N. Wang K. L. C. Hussey P. J.. (2006). The POLARIS peptide of Arabidopsis regulates auxin transport and root growth via effects on ethylene signaling. Plant Cell 18, 3058–3072. 10.1105/tpc.106.040790 17138700 PMC1693943

[B17] Chu Q. Rathore A. Diedrich J. K. Donaldson C. J. Yates J. R. III. Saghatelian A. (2017). Identification of microprotein-protein interactions via apex tagging. Biochemistry 56, 3299–3306. 10.1021/acs.biochem.7b00265 28589727 PMC5499098

[B18] Cohen S. M. (2014). Everything old is new again: (linc) RNAs make proteins! EMBO J. 33, 937–938. 10.1002/embj.201488303 24719208 PMC4193927

[B19] Crappé J. Van Criekinge W. Menschaert G. (2014). Little things make big things happen: a summary of micropeptide encoding genes. EuPA Open Proteomics 3, 128–137. 10.1016/j.euprot.2014.02.006

[B20] Cunha F. M. Berti D. A. Ferreira Z. S. Klitzke C. F. Markus R. P. Ferro E. S. (2008). Intracellular peptides as natural regulators of cell signaling. J. Biol. Chem. 283, 24448–24445. 10.1074/jbc.M801252200 18617518 PMC3259820

[B21] D'Lima N. G. Ma J. Winkler L. Chu Q. Loh K. H. Corpuz E. O.. (2017). A human microprotein that interacts with the mRNA decapping complex. Nat. Chem. Biol. 13, 174–180. 10.1038/nchembio.2249 27918561 PMC5247292

[B22] Dong X. Wang D. Liu P. Li C. Zhao Q. Zhu D.. (2013). Zm908p11, encoded by a short open reading frame (sORF) gene, functions in pollen tube growth as a profilin ligand in maize. J. Exp. Bot. 64, 2359–2372. 10.1093/jxb/ert093 23676884 PMC3654424

[B23] Fields A. P. Rodriguez E. H. Jovanovic M. Stern-Ginossar N. Haas B. J. Mertins P.. (2015). A regression-based analysis of ribosome-profiling data reveals a conserved complexity to mammalian translation. Mol. Cell 60, 816–827. 10.1016/j.molcel.2015.11.013 26638175 PMC4720255

[B24] Frank M. J. Smith L. G. (2002). A small, novel protein highly conserved in plants and animals promotes the polarized growth and division of maize leaf epidermal cells. Curr. Biol. 12, 849–853. 10.1016/S0960-9822(02)00819-9 12015123

[B25] Fricker L. D. (2005). Neuropeptide-processing enzymes: applications for drug discovery. AAPS J. 7, E449–E455. 10.1208/aapsj070244 16353923 PMC2750981

[B26] Frith M. C. Forrest A. R. Nourbakhsh E. Pang K. C. Kai C. Kawai J.. (2006). The abundance of short proteins in the mammalian proteome. PLoS Genet. 2:e52. 10.1371/journal.pgen.0020052 16683031 PMC1449894

[B27] Galindo M. I. Pueyo J. I. Fouix S. Bishop S. A. Couso J. P. (2007). Peptides encoded by short ORFs control development and define a new eukaryotic gene family. PLoS Biol. 5:e106. 10.1371/journal.pbio.0050106 17439302 PMC1852585

[B28] Gish W. States D. J. (1993). Identification of protein coding regions by database similarity search. Nat. Genet. 3, 266–272. 10.1038/ng0393-266 8485583

[B29] Grundy G. J. Rulten S. L. Arribas-Bosacoma R. Davidson K. Kozik Z. Oliver A. W.. (2016). The Ku-binding motif is a conserved module for recruitment and stimulation of non-homologous end-joining proteins. Nat. Commun. 7:11242. 10.1038/ncomms11242 27063109 PMC4831024

[B30] Guo B. Zhai D. Cabezas E. Welsh K. Nouraini S. Satterthwait A. C.. (2003). Humanin peptide suppresses apoptosis by interfering with Bax activation. Nature 423, 456–461. 10.1038/nature01627 12732850

[B31] Guo P. Yoshimura A. Ishikawa N. Yamaguchi T. Guo Y. Tsukaya H. (2015). Comparative analysis of the RTFL peptide family on the control of plant organogenesis. J. Plant Res. 128, 497–510. 10.1007/s10265-015-0703-1 25701405 PMC4408365

[B32] Guttman M. Russell P. Ingolia N. T. Weissman J. S. Lander E. S. (2013). Ribosome profiling provides evidence that large noncoding RNAs do not encode proteins. Cell 154, 240–251. 10.1016/j.cell.2013.06.009 23810193 PMC3756563

[B33] Hanada K. Akiyama K. Sakurai T. Toyoda T. Shinozaki K. Shiu S. H. (2010). sORF finder: a program package to identify small open reading frames with high coding potential. Bioinformatics 26, 399–400. 10.1093/bioinformatics/btp688 20008477

[B34] Hanada K. Higuchi-Takeuchi M. Okamoto M. Yoshizumi T. Shimizu M. Nakaminami K.. (2013). Small open reading frames associated with morphogenesis are hidden in plant genomes. Proc. Natl. Acad. Sci. U.S.A. 110, 2395–2400. 10.1073/pnas.1213958110 23341627 PMC3568369

[B35] Hashimoto Y. Niikura T. Tajima H. Yasukawa T. Sudo H. Ito Y.. (2001). A rescue factor abolishing neuronal cell death by a wide spectrum of familial Alzheimer's disease genes and Abeta. Proc. Natl. Acad. Sci. U.S.A. 98, 6336–6341. 10.1073/pnas.101133498 11371646 PMC33469

[B36] Housman G. Ulitsky I. (2016). Methods for distinguishing between protein-coding and long noncoding RNAs and the elusive biological purpose of translation of long noncoding RNAs. Biochim. Biophys. Acta 1859, 31–40. 10.1016/j.bbagrm.2015.07.017 26265145

[B37] Huang J. Z. Chen M. Chen D. Gao X. C. Zhu S. Huang H.. (2017). A peptide encoded by a putative lncRNA HOXB-AS3 suppresses colon cancer growth. Mol. Cell 68, 171–184. 10.1016/j.molcel.2017.09.015 28985503

[B38] Ingolia N. T. Brar G. A. Stern-Ginossar N. Harris M. S. Talhouarne G. J. Jackson S. E.. (2014). Ribosome profiling reveals pervasive translation outside of annotated protein-coding genes. Cell Rep. 8, 1365–1379. 10.1016/j.celrep.2014.07.045 25159147 PMC4216110

[B39] Ingolia N. T. Lareau L. F. Weissman J. S. (2011). Ribosome profiling of mouse embryonic stem cells reveals the complexity and dynamics of mammalian proteomes. Cell 147, 789–802. 10.1016/j.cell.2011.10.002 22056041 PMC3225288

[B40] Kapranov P. Willingham A. T. Gingeras T. R. (2007). Genome-wide transcription and the implications for genomic organization. Nat. Rev. Genet. 8, 413–423. 10.1038/nrg2083 17486121

[B41] Kikuchi K. Fukuda M. Ito T. Inoue M. Yokoi T. Chiku S.. (2009). Transcripts of unknown function in multiple-signaling pathways involved in human stem cell differentiation. Nucleic Acids Res. 37, 4987–5000. 10.1093/nar/gkp426 19531736 PMC2731886

[B42] Kochetov A. V. (2005). AUG codons at the beginning of protein coding sequences are frequent in eukaryotic mRNAs with a suboptimal start codon context. Bioinformatics 21, 837–840. 10.1093/bioinformatics/bti136 15531618

[B43] Kondo T. Hashimoto Y. Kato K. Inagaki S. Hayashi S. Kageyama Y. (2007). Small peptide regulators of actin-based cell morphogenesis encoded by a polycistronic mRNA. Nat. Cell Biol. 9, 660–665. 10.1038/ncb1595 17486114

[B44] Kondo T. Plaza S. Zanet J. Benrabah E. Valenti P. Hashimoto Y.. (2010). Small peptides switch the transcriptional activity of Shavenbaby during *Drosophila embryogenesis*. Science 329, 336–339. 10.1126/science.1188158 20647469

[B45] Ladoukakis E. Pereira V. Magny E. G. Eyre-Walker A. Couso J. P. (2011). Hundreds of putatively functional small open reading frames in Drosophila. Genome Biol. 12:R118. 10.1186/gb-2011-12-11-r118 22118156 PMC3334604

[B46] Lauressergues D. Couzigou J. M. San Clemente H. Martinez Y. Dunand C. Bécard G.. (2015). Primary transcripts of microRNAs encode regulatory peptides. Nature 520, 90–93. 10.1038/nature14346 25807486

[B47] Lee C. Zeng J. Drew B. G. Sallam T. Martin-Montalvo A. Wan J.. (2015). The mitochondrial-derived peptide MOTS-c promotes metabolic homeostasis and reduces obesity and insulin resistance. Cell Metab. 21, 443–454. 10.1016/j.cmet.2015.02.009 25738459 PMC4350682

[B48] Lin M. F. Jungreis I. Kellis M. (2011). PhyloCSF: a comparative genomics method to distinguish protein coding and non-coding regions. Bioinformatics 27, i275–i282. 10.1093/bioinformatics/btr209 21685081 PMC3117341

[B49] Ma J. Ward C. C. Jungreis I. Slavoff S. A. Schwaid A. G. Neveu J.. (2014). Discovery of human sORF-encoded polypeptides (SEPs) in cell lines and tissue. J. Proteome Res. 13, 1757–1765. 10.1021/pr401280w 24490786 PMC3993966

[B50] Ma J. Yan B. Qu Y. Qin F. Yang Y. Hao X.. (2008). Zm401, a short-open reading-frame mRNA or noncoding RNA, is essential for tapetum and microspore development and can regulate the floret formation in maize. J. Cell. Biochem. 105, 136–146. 10.1002/jcb.21807 18465785

[B51] Mackowiak S. D. Zauber H. Bielow C. Thiel D. Kutz K. Calviello L.. (2015). Extensive identification and analysis of conserved small ORFs in animals. Genome Biol. 16:179. 10.1186/s13059-015-0742-x 26364619 PMC4568590

[B52] Magny E. G. Pueyo J. I. Pearl F. M. Cespedes M. A. Niven J. E. Bishop S. A.. (2013). Conserved regulation of cardiac calcium uptake by peptides encoded in small open reading frames. Science 341, 1116–1120. 10.1126/science.1238802 23970561

[B53] Makarewich C. A. Olson E. N. (2017). Mining for micropeptides. Trends Cell Biol. 27, 685–696. 10.1016/j.tcb.2017.04.006 28528987 PMC5565689

[B54] Matsumoto A. Pasut A. Matsumoto M. Yamashita R. Fung J. Monteleone E.. (2017). mTORC1 and muscle regeneration are regulated by the LINC00961-encoded SPAR polypeptide. Nature 541, 228–232. 10.1038/nature21034 28024296

[B55] Matsuoka M. Hashimoto Y. Aiso S. Nishimoto I. (2006). Humanin and colivelin: neuronal-death-suppressing peptides for Alzheimer's disease and amyotrophic lateral sclerosis. CNS Drug Rev. 12, 113–122. 10.1111/j.1527-3458.2006.00113.x 16958985 PMC6494132

[B56] Narita N. N. Moore S. Horiguchi G. Kubo M. Demura T. Fukuda H.. (2004). Overexpression of a novel small peptide ROTUNDIFOLIA4 decreases cell proliferation and alters leaf shape in *Arabidopsis thaliana*. Plant J. 38, 699–713. 10.1111/j.1365-313X.2004.02078.x 15125775

[B57] Nelson B. R. Makarewich C. A. Anderson D. M. Winders B. R. Troupes C. D. Wu F.. (2016). A peptide encoded by a transcript annotated as long noncoding RNA enhances SERCA activity in muscle. Science 351, 271–275. 10.1126/science.aad4076 26816378 PMC4892890

[B58] Pauli A. Norris M. L. Valen E. Chew G. L. Gagnon J. A. Zimmerman S.. (2014). Toddler: an embryonic signal that promotes cell movement via Apelin receptors. Science 343:1248636. 10.1126/science.1248636 24407481 PMC4107353

[B59] Pauli A. Valen E. Schier A. F. (2015). Identifying (non-)coding RNAs and small peptides: challenges and opportunities. Bioessays 37, 103–112. 10.1002/bies.201400103 25345765 PMC4433033

[B60] Razooky B. S. Obermayer B. O'May J. B. Tarakhovsky A. (2017). Viral infection identifies micropeptides differentially regulated in smORF-containing lncRNAs. Genes 8:206. 10.3390/genes8080206 28825667 PMC5575669

[B61] Rhee H. W. Zou P. Udeshi N. D. Martell J. D. Mootha V. K. Carr S. A.. (2013). Proteomic mapping of mitochondria in living cells via spatially restricted enzymatic tagging. Science 339, 1328–1331. 10.1126/science.1230593 23371551 PMC3916822

[B62] Rohrig H. Schmidt J. Miklashevichs E. Schell J. John M. (2002). Soybean ENOD40 encodes two peptides that bind to sucrose synthase. Proc. Natl. Acad. Sci. U.S.A. 99, 1915–1920. 10.1073/pnas.022664799 11842184 PMC122294

[B63] Saghatelian A. Couso J. P. (2015). Discovery and characterization of smORF-encoded bioactive polypeptides. Nat. Chem. Biol. 11:909. 10.1038/nchembio.1964 26575237 PMC4956473

[B64] Savard J. Marques-Souza H. Aranda M. Tautz D. (2006). A segmentation gene in tribolium produces a polycistronic mRNA that codes for multiple conserved peptides. Cell 126, 559–569. 10.1016/j.cell.2006.05.053 16901788

[B65] Schwaid A. G. Shannon D. A. Ma J. Slavoff S. A. Levin J. Z. Weerapana E.. (2013). Chemoproteomic discovery of cysteine-containing human short open reading frames. J. Am. Chem. Soc. 135, 16750–16753. 10.1021/ja406606j 24152191 PMC3868496

[B66] Siepel A. Bejerano G. Pedersen J. S. Hinrichs A. S. Hou M. Rosenbloom K.. (2005). Evolutionarily conserved elements in vertebrate, insect, worm, and yeast genomes. Genome Res. 15, 1034–1050. 10.1101/gr.3715005 16024819 PMC1182216

[B67] Skarshewski A. Stanton-Cook M. Huber T. Al Mansoori S. Smith R. Beatson S. A.. (2014). uPEPperoni: an online tool for upstream open reading frame location and analysis of transcript conservation. BMC Bioinformatics 15:36. 10.1186/1471-2105-15-36 24484385 PMC3914846

[B68] Slavoff S. A. Heo J. Budnik B. A. Hanakahi L. A. Saghatelian A. (2014). A human short open reading frame (sORF)-encoded polypeptide that stimulates DNA end joining. J. Biol. Chem. 289, 10950–10957. 10.1074/jbc.C113.533968 24610814 PMC4036235

[B69] Slavoff S. A. Mitchell A. J. Schwaid A. G. Cabili M. N. Ma J. Levin J. Z.. (2013). Peptidomic discovery of short open reading frame-encoded peptides in human cells. Nat. Chem. Biol. 9, 59–64. 10.1038/nchembio.1120 23160002 PMC3625679

[B70] Smith J. E. Alvarez-Dominguez J. R. Kline N. Huynh N. J. Geisler S. Hu W.. (2014). Translation of small open reading frames within unannotated RNA transcripts in *Saccharomyces cerevisiae*. Cell Rep. 7, 1858–1866. 10.1016/j.celrep.2014.05.023 24931603 PMC4105149

[B71] Valdivia E. R. Chevalier D. Sampedro J. Taylor I. Niederhuth C. E. Walker J. C. (2012). DVL genes play a role in the coordination of socket cell recruitment and differentiation. J. Exp. Bot. 63, 1405–1412. 10.1093/jxb/err378 22112938 PMC3276101

[B72] Vanderperre B. Lucier J. F. Roucou X. (2012). HAltORF: a database of predicted out-of-frame alternative open reading frames in human. Database 2012:bas025. 10.1093/database/bas025 22613085 PMC3356836

[B73] Wang D. Li C. Zhao Q. Zhao L. Wang M. Zhu D. (2009). Zm401p10, encoded by an anther-specific gene with short open reading frames, is essential for tapetum degeneration and anther development in maize. Funct. Plant Biol. 36, 73–85. 10.1071/FP08154 32688629

[B74] Wen J. Lease K. A. Walker J. C. (2004). DVL, a novel class of small polypeptides: overexpression alters Arabidopsis development. Plant J. 37, 668–677. 10.1111/j.1365-313X.2003.01994.x 14871303

[B75] Willingham A. T. Dike S. Cheng J. Manak J. R. Bell I. Cheung E.. (2006). Transcriptional landscape of the human and fly genomes: nonlinear and multifunctional modular model of transcriptomes. Cold Spring Harb. Symp. Quant. Biol. 71, 101–110. 10.1101/sqb.2006.71.068 17480199

[B76] Zanet J. Benrabah E. Li T. Pelissier-Monier A. Chanut-Delalande H. Ronsin B.. (2015). Pri sORF peptides induce selective proteasome-mediated protein processing. Science 349, 1356–1358. 10.1126/science.aac5677 26383956

[B77] Zhang Q. Vashisht A. A. O'Rourke J. Corbel S. Y. Moran R. Romero A.. (2017). The microprotein Minion controls cell fusion and muscle formation. Nat. Commun. 8:15664. 10.1038/ncomms15664 28569745 PMC5461507

